# Paine’s Institutes of Medicine

**Published:** 1859-12

**Authors:** 


*Paine's Institutes of Medicine.*—We have received, through the kindness of the author, the fifth edition of Paine's Institutes of Medicine, published by the Harpers, of this city. An elaborate review of the fourth edition of this work appeared in the *Journal* but little more than a year ago, from the pen of one much better qualified to do the subject justice than ourselves. The appearance of another edition so soon must be gratifying to the author. The new edition contains some new corrections and references, and a supplement.

